# Paroxysmal Movement Disorders

**DOI:** 10.3389/fneur.2021.659064

**Published:** 2021-06-11

**Authors:** Susan Harvey, Mary D. King, Kathleen M. Gorman

**Affiliations:** ^1^Department of Paediatric Neurology and Clinical Neurophysiology, Children's Health Ireland at Temple Street, Dublin, Ireland; ^2^School of Medicine and Medical Science, University College Dublin, Dublin, Ireland

**Keywords:** paroxysmal movement disorders, genetics, next-generation sequencing, paroxysmal dyskinesia, episodic ataxia

## Abstract

Paroxysmal movement disorders (PxMDs) are a clinical and genetically heterogeneous group of movement disorders characterized by episodic involuntary movements (dystonia, dyskinesia, chorea and/or ataxia). Historically, PxMDs were classified clinically (triggers and characteristics of the movements) and this directed single-gene testing. With the advent of next-generation sequencing (NGS), how we classify and investigate PxMDs has been transformed. Next-generation sequencing has enabled new gene discovery (*RHOBTB2, TBC1D24*), expansion of phenotypes in known PxMDs genes and a better understanding of disease mechanisms. However, PxMDs exhibit phenotypic pleiotropy and genetic heterogeneity, making it challenging to predict genotype based on the clinical phenotype. For example, paroxysmal kinesigenic dyskinesia is most commonly associated with variants in *PRRT2* but also variants identified in *PNKD, SCN8A, and SCL2A1*. There are no radiological or biochemical biomarkers to differentiate genetic causes. Even with NGS, diagnosis rates are variable, ranging from 11 to 51% depending on the cohort studied and technology employed. Thus, a large proportion of patients remain undiagnosed compared to other neurological disorders such as epilepsy, highlighting the need for further genomic research in PxMDs. Whole-genome sequencing, deep-sequencing, copy number variant analysis, detection of deep-intronic variants, mosaicism and repeat expansions, will improve diagnostic rates. Identifying the underlying genetic cause has a significant impact on patient care, modification of treatment, long-term prognostication and genetic counseling. This paper provides an update on the genetics of PxMDs, description of PxMDs classified according to causative gene rather than clinical phenotype, highlighting key clinical features and providing an algorithm for genetic testing of PxMDs.

## Introduction

Paroxysmal movement disorders (PxMDs) are a clinical and genetically heterogeneous group characterized by episodic involuntary movements (dystonia, dyskinesia, chorea and/or ataxia). Historically, PxMDs were characterized clinically by the age of onset, triggers, duration and predominant movements (1, 2). Paroxysmal movement disorders are divided into paroxysmal dyskinesia or episodic ataxia (EA) depending on the main movement. However, both may co-exist with additional co-morbidities (epilepsy, headache, etc.). In both subgroups, symptoms begin in childhood or adolescence and improve or even resolve with age. Typically, between the events, neurological examination is normal. Sometimes in EA, chronic ataxia or nystagmus may co-exist. Although PxMDs can be due to secondary or acquired causes such a structural, immunological (multiple sclerosis), metabolic or neurodegenerative disorder, this review will focus on the genetic causes of PxMDs, previously referred to as primary PxMDs. Paroxysmal movement disorders are considered to be due to disruption of the network between cerebellar output, striatal dysfunction or a combination (3).

Before the era of next-generation sequencing (NGS), the clinical phenotype directed single gene testing for PxMDs. Most commonly testing of *PRRT2* for paroxysmal kinesigenic dyskinesia (PKD), *PNKD* in paroxysmal non-kinesigenic dyskinesia (PNKD) or *SLC2A1* in paroxysmal-exercised induced dyskinesia (PED). However, a large number (~50%) remained without a genetic cause, despite exhibiting the classical features of PxMDs (4). The advent of NGS has enabled the discovery of new genes associated with PxMDs (*RHOBTB2, TBC1D24, PDE2A*) and the expansion of the phenotypes of known PxMDs (5–8). For example, pathogenic variants in *PRRT2* are now associated with PKD, PNKD, PED, hemiplegic migraine and self-limiting infantile seizures (9–11). Thus, PxMDs exhibit significant phenotypic pleiotropy and genetic heterogeneity, making it challenging to predict genotype based on the clinical phenotype.

With the identification of new phenotypes and new genes, the limits of the current classification system have become apparent. Sole identification of a trigger or the predominant movement disorder are no longer sufficient to predict the genotype. Various re-organizations have been introduced to alleviate the confusion of presentations of both adult and pediatric PxMDs (12). These systems aim to provide an accurate and criterion-based list of genetically confirmed disorders in which the naming system conveys the causative gene and maintains the connection between the phenotype and the gene. For example, Marras and colleagues suggest PxMD-*PRRT2* for PKD caused by *PRRT2* variants, clearly outlining its episodic nature along with the genetic cause (12). This updated system mirrors the evolution of the classification of epilepsy syndromes to now reflect the gene rather than the clinical or electroencephalogram features (13).

This paper provides an update on the genetics of PxMDs, classified according to the gene involved, highlights the key clinical features and provides an algorithm for genetic testing of PxMDs in the era of NGS.

## Predominant Dyskinesia Genes

Paroxysmal dyskinesias are characterized by recurrent attacks of involuntary movement (chorea and/or dystonia often with ballismus and athetosis) without loss of consciousness. They are subdivided into three main groups depending on the key trigger for movements: sudden voluntary movements (PKD), no clear trigger (PNKD), or prolonged-exercise (PED) (1, 2). Recent reviews have provided excellent summaries on the clinical presentation, the evolution of nomenclature and terminology and treatment (2, 4). Table 1 provides a list of predominant dyskinesia genes organized according to function detailing inheritance, phenotypes and potential treatments. We have chosen to discuss eight dyskinesia genes in detail on the basis of high prevalence in clinical practice in the case of five genes (*PRRT2, PNKD, SLC2A1, KCNMA1, ADCY5*) and the recent discovery and newly described phenotypes (*RHOBTB2, TBC1D24, PDE2A*).

**Table 1:** Predominant dyskinesia genes.

**Gene (most common in bold)**	**Cytogenic location**	**Inheritance**	**Paroxysmal MD (most common MD in bold)**	**Other phenotypes**	**Potential treatment**	**References**
**Neurotransmission and synaptopathies**						
*CHRNA4*	20q13.33	AD	PKD	Epilepsy	OXY	(14, 15)
*FGF14*	13q33.1	AD	**EA** PNKD PKD	ID SCA 27	ACZ	(16–21)
*GCH1*	14q22.2	AD	PED	Dopa-responsive dystonia	Levodopa	(22)
*PARK2*	6q26	AR	PED	Juvenile-onset Parkinson Disease	–	(23, 24)
***PNKD***	2q35	AD	**PNKD** PKD PED	FHM Hemiplegic episodes	BZ LEV VPA DBS	(25, 26)
***PRRT2***	16p11.2	AD/AR	**PKD** **PKD** **+** **Neonatal seizures** PED PNKD Paroxysmal torticollis EA	Absence epilepsy DEE FHM ID Self-limiting neonatal seizures	CBZ (PKD) ACZ (EA)	(4, 8, 27–37)
*RHOBTB2*	8p21.3	AD	PKD EA AHC-like episodes	DEE Mixed MD	CBZ	(5, 38–40)
*TBC1D24*	16p13.3	AR	**PED**	DEE Deafness DOORS EPC Polymyoclonus	CBZ DPM ACZ	(6, 41–43)
**Channelopathies**						
*ATP1A2*	1q23.2	AD	**PNKD**	FHM	–	(18, 44)
*ATP1A3*	19q13.2	AD	**PNKD** PED Paroxysmal dystonia Paroxysmal ataxia EA	AHC CAPOS DEE Fever-triggered encephalopathy Rapid-onset Dystonia Parkinsonism	FLUN KD	(23, 45, 46)
*CACNA1A*	19p13.13	AD	PNKD PED Paroxysmal torticollis Paroxysmal tonic up-gaze **EA**	Congenital cerebellar ataxia DEE Epilepsy FHM Hemiplegic episodes Nystagmus SCA6	ACZ 4-AP VPA FLUN	(47–54)
***KCNMA1***	10q22.3	AD	PNKD	Developmental delay Epilepsy Liang-Wang Syndrome	–	(55–59)
*SCN8A*	12q13.13	AD	PKD PKD + Neonatal seizures	DEE	CBZ OXY	(60–62)
**cAMP/cGMP – Phosphodiesterases**						
***ADCY5***	3q21.1	AD	Nocturnal PxMDs PKD PED PNKD Facial dyskinesia & orofacial jerks AHC	Mixed MD: chorea, dystonia or myoclonus Axial hypotonia Developmental delay Myoclonus dystonia	Caffeine ACZ DBS Clonazepam	(23, 63–68)
*DEPDC5*	22q12.2	AD	PKD	Focal epilepsy	CBZ	(69)
*GNAO1*	16q13	AD	PNKD	DEE ID	DBS	(70)
*PDE2A*	11q13.4	AR	PKD	Chorea Epilepsy ID	Nil	(7)
**Brain energy transportopathies**						
***SLC2A1***	1p34.2	AD	**PED** PNKD PKD PED + spasticity PED + epilepsy EA	DEE Epilepsy/absence epilepsy FHM ID Spastic paraplegia	KD Trihepantoin	(4, 23, 71–77)
*BCKD complex*	19q13.2/6q14.1	AR	PNKD EA	MSUD	Isoleucine & valine restriction	(78)
*ECHS1*	10q.26.3	AR	**PED** (± bilateral T2 hyperintensities in globus pallidi) PNKD	Epilepsy ID Leigh syndrome	KD Mitochondrial cocktail	(79, 80)
*GLDC*	9p24.1	AR	PNKD	Non-ketotic hyperglycinemia	Sodium benzoate, KD	(81)
**Pyruvate dehydrogenase complex**						
*PDHA1*	Xp22.12	X-linked	PED PNKD Paroxysmal dystonia EA	Neonatal lactic acidosis Epilepsy Hypotonia Leigh Syndrome	Thiamine (PED/EA) Levodopa KD (Dystonia)	(82–85)
*PDHX*	11p13	AR				
*DLAT*	11q23.1	AR				
**Other**						
*SLC20A2*	8p11.21	AD	**PNKD** PKD	Familial idiopathic basal ganglia calcification	CBZ	(86)

*ACZ, Acetazolamide; AD, Autosomal Dominant; AR, Autosomal recessive; AHC, Alternating hemiplegia of childhood; CAPOS, Cerebellar ataxia, areflexia, pes cavus, optic atrophy and sensorineural hearing loss; CBZ, Carbamazepine; DBS, Deep-brain Stimulation; DEE, Developmental and epileptic encephalopathy; DPM, Diazepam; EA, Episodic Ataxia; FHM, Familial Hemiplegic Migraine; FLUN, Flunarizine; ID, Intellectual Disability; KD, Ketogenic diet, LEV, Levetiracetam; MD, Movement Disorder; MSUD, Maple-syrup urine disease; OXY, Oxcarbazepine; PED, Paroxysmal Exercise Induced Dyskinesia; PKD, Paroxysmal Kinesigenic Dyskinesia; PMD, Paroxysmal movement disorder; PNKD, Paroxysmal Non-Kinesigenic Dyskinesia; SCA, Spinocerebellar ataxia; VPA, Sodium Valproate; 4-AP, 4-aminopyridine*.

### *PRRT2* (Chr 16:29,812,192-29,815,919, MIM: 614386)

Proline-rich transmembrane protein 2 (*PRRT2)* is a transmembrane protein which interacts with pre-synaptic proteins (SNAP25, VAMP2, SYT1, and SYT2) and affects neurotransmitter release. Initially reported as the first causative gene in PKD in 2011, pathogenic variants in *PRRT2* are now implicated in various pediatric disorders with marked phenotypic pleiotropy (9–11).

Loss-of-function (LOF) variants in *PRRT2* are the most common cause of PKD, accounting for 27–65% of all cases (25, 27). Symptoms typically begin in childhood or early adolescence [mean age of onset of 9.9 years (range: 1–40 years)]. Events are characterized by brief daily episodes of dystonia (17.6%), chorea (15.2%) or both (67.1%) which are triggered by voluntary movements that are often sudden (8). Of note, attacks can be triggered by coffee, anxiety or sleep deprivation in 40% in keeping with a PNKD phenotype. In a subset, neonatal or early-onset seizures occur (previously called infantile convulsions with choreoathetosis), with PKD emerging later, and is now viewed as part of PKD rather than a separate disorder. It occurs in 30% of *PRRT2*-associated PKD and is referred to as Paroxysmal Kinesigenic Dyskinesia with infantile Convulsions (PKD/IC) (8).

Other paroxysmal disorders associated with *PRRT2* include: PED, PNKD, paroxysmal torticollis, EA and familial hemiplegic migraine (FHM), which may co-exist with PKD or occur independently (4, 27–29). Also, pathogenic variants in *PRRT2* are reported in febrile seizures and less commonly in childhood absence epilepsy in the absence of PKD (30, 87).

The majority of cases have an autosomal dominant (AD) inheritance with incomplete penetrance reported (60–90%) (8, 31). The c.649dupC is a recurrent variant reported in 53.7% of PKD cases (with or without neonatal seizures). However, biallelic variants are also reported with a more severe phenotype of PKD, self-limiting neonatal seizures, intellectual disability (ID), EA and absence epilepsy (32, 33).

### *PNKD* (Chr 2:218,270,484-218,346,792, MIM:609023)

Metallo-beta-lactamase domain-containing protein (*PNKD*) has multiple alternative names: myofibrillogenesis regulator 1 (*MR-1*), transactivated by hepatitis C virus core protein 2 (*TAHCCP2*), brain protein 17 (*BRP17*) and *KIAA1184*. Located in the cell membrane, and specifically expressed in the brain, *PNKD* was initially identified in the stress response pathway, and recent work suggests a role in maintaining cellular redox status (88, 89).

Pathogenic variants in *PNKD* account for 70% of PNKD (25). There are two recurrent variants p.Ala7Val and p.Ala9Val with an AD pattern of inheritance and almost complete penetrance (95%) (4, 26, 90). Compared to *PRRT2* or *SLC2A1*, PNKD is not associated with significant pleiotropy, although a single family with FHM and no PxMD is published (4). In *PNKD*, attacks consisting of chorea and/or dystonia triggered by stress, alcohol, coffee, tea or strong emotion (laughing, excitement etc.) occur. Episodes are longer than in PKD, typically between 10 min and 1 h but can last up to 12 h and are infrequent occurring only a few times per year. Onset is from childhood to early adolescence [mean age of onset 5 years (range: 6 months–35 years)] with a reduction in the frequency of attacks in adulthood (23, 55, 91). There is no associated epilepsy or neurodevelopmental disorder, in contrast to the PNKD phenotype associated with *KCNMA1*.

### *SLC2A1* (Chr 1:42,925,352-42,958,867, MIM:138140)

Solute carrier family 2 member 1 (*SLC2A1*) codes for glucose transporter-1 (GLUT-1), the major glucose transporter in the blood-brain barrier. Disruption of GLUT-1 causes a reduction in glucose available to the brain. Variants in *SCL2A1* are responsible for a broad spectrum of neurological disorders including PxMDs (PED, PKD, EA), non-PxMDs (chorea/dystonia), chronic ataxia, classic GLUT-1 deficiency syndrome (developmental delay, hypotonia, postnatal microcephaly, epilepsy), ID, refractory absence epilepsy, FHM, and hereditary spastic paraplegia (4, 71–74).

Most variants occur *de novo* or are inherited from a symptomatic parent, but autosomal recessive (AR) inheritance has been published (92, 93). Genotype-phenotype correlations are observed with LOF variants (splicing, protein-truncating variants, insertions/deletions) presenting with an earlier onset and more severe presentation. Missense variants (20%) are associated with milder symptoms, as there is larger residual activity of GLUT1. However, the same variant in *SLC2A1* can be implicated in different clinical phenotypes, for example, p.Arg126Cys is reported in hereditary spastic paraplegia, classic GLUT-1 deficiency and early-onset absence epilepsy. Therefore, other as yet unknown factors, such as epigenetic modifiers or environmental effects, are likely to play a role (75).

Loss-of-function variants in the *SLC2A1* account for 20% of PED characterized by dystonia and/or chorea after prolonged exercise and fasting (4, 94). Attack duration (minutes-hours) is variable, as is the level of physical activity required to trigger an attack (23). The mean age of symptom onset in PED is 8.6 years (range: 1–49 years) (25). Paroxysmal-exercised induced dyskinesia may be associated with a slowly progressive paraparesis or generalized epilepsy (4, 75, 76).

### *KCNMA1* (Chr 10:76,869,601-77,637,968, MIM: 600150)

The alpha, pore-forming subunit of the calcium-sensitive (BK) channel (K_Ca_1.1) is important for neuronal excitability and is encoded by potassium calcium-activated channel subfamily M Alpha 1 (*KCNMA1*) (91). The core features of *KCNMA1*-related disorders (both GOF and LOF) are epilepsy, movement disorders, neurodevelopmental disorders and ID. The majority of pathogenic variants are *de novo*; however, AD and AR inheritance are reported (55, 56, 91). There is intrafamilial phenotypic variability and incomplete penetrance. The movement disorder phenotype can be predicted by the functional changes in BK activity. Paroxysmal non-kinesigenic dyskinesia is associated with GOF variants and ataxia and tremor with LOF variants (91).

The initial report of pathogenic variants in *KCNMA1* described the largest cohort of affected patients (15 family members) with p.Asp434Gly with a variable phenotype of PNKD and/or epilepsy. Two recurrent GOF variants (p.Asp434Gly and p.Asn1053Ser) are associated with PNKD and epilepsy (91). The mean age of onset of PxMD is 4.6 years (range: 1–15 years) (25). Overlapping with *PNKD*, alcohol is a trigger for some (57). Developmental delay and epilepsy frequently co-exist, in contrast to PxMD-*PNKD* (58, 95).

A recurrent LOF variant (p.Gly375Arg) is associated with a distinct neurodevelopmental disorder (Liang-Wang Syndrome, MIM:618729) of developmental delay, seizures, dysmorphic features, visceral and cardiac malformations but without movement disorder (59). Other LOF variants are described in neurodevelopmental disorders, cerebral atrophy, ID and ataxia (59). Homozygous variants in *KCNMA1* are published in two consanguineous families with LOF variants and clinical phenotype of cerebellar atrophy, developmental delay and seizures (55, 56).

### *ADCY5* (Chr 3:123,282,295-123,449,089, MIM: 600293)

Adenylate cyclase 5 (*ADCY5)* is necessary to convert adenosine triphosphate to cyclic adenosine 3'5' monophosphate (cAMP), a secondary messenger for multiple cellular activities. The high expression of ADCY5 in the striatum has led to the hypothesis that altered dopamine signaling in response to stress is responsible for *ADCY5-*related dyskinesia (96). There is phenotypic variability which appears dependent on the gene domain affected. Pathogenic variants in the intracellular catalytic cyclase domains C1a and C2a are associated with moderate to severe presentations, compared to a milder phenotype and no hypotonia with variants in the second portion of the first cytoplasmic domain (C1b) (96). The p.Arg413 hotspot (p.Arg416Gln/Gly/Trp), accounts for 50% of cases (96, 97). The majority of pathogenic variants are *de novo*. Mosaicism is observed frequently (25–50% of cases) and associated with a milder phenotype (96, 98). Two families with biallelic variants are described (99).

Pathogenic variants in *ADCY5* are associated with a childhood-onset of mixed hyperkinetic movement disorder (chorea, dystonia or myoclonus or a combination) with orofacial jerks (facial myokymia), oculomotor apraxia and sleep-related PxMD (98). The nocturnal dyskinesia, in particular, appears characteristic and distinguishes it from most other movement disorders, in which sleep abolishes dyskinesia. The abnormal nocturnal movements occur more frequently in stage two and rapid-eye-movement sleep and have a significant negative impact on the quality of sleep (100, 101). Although some experience continuous symptoms, paroxysmal exacerbations of hyperkinetic movements are common, often triggered by illness, emotions or stress. Exacerbations may last for minutes to hours, sometimes referred to as “*ballistic bouts.”* Caffeine may exacerbate PxMD in some, but is an effective treatment in others (63). The movement disorder is usually static or very slowly progressive and can improve with age. Hypotonia and motor delay may predate the onset of movement disorder (97, 98). Less common phenotypes include alternating hemiplegia of childhood (AHC), myoclonus-dystonia, isolated dystonia and childhood-onset chorea (96). Thus, the range of movement disorders reported with pathogenic variants in *ADCY5* is expanding and other PxMDs phenotypes may emerge.

### *RHOBTB2* (Chr 8:22,987,250-23,020,198, MIM: 607352)

Heterozygous missense variants in Rho-related BTB domain-containing protein 2 (*RHOBTB2*) were reported in 2018 in 13 individuals with DEEs (102, 103). In the initial reports, 11/13 had a movement disorder (dystonia, dyskinesia, chorea and stereotypies), described as paroxysmal in 4/11 (paroxysmal dyskinesia, dystonic-athetoid attacks) (5, 38, 103). Subsequently, two case reports focusing on the PxMDs have been published, including a patient with paroxysmal dyskinesia and developmental delay without epilepsy (38, 39). Recently a cohort of 11 patients was published with a complex, polymorphic movement disorder, with both paroxysmal and non-paroxysmal features (40). Paroxysmal movements were characterized by hemiplegic episodes, focal dystonia, worsening dyskinesia, episodic ataxia, and non-epileptic myoclonus. Recurrent variants are reported (p.Arg483His, p.Arg511Gln), but numbers are too small to identify any genotype-phenotype correlations. All variants reported to date are *de novo*. Thus, further reports will help to elucidate the full spectrum of *RHOBTB2*-related movement disorder (paroxysmal and non-paroxysmal).

### *TBC1D24* (Chr 16:2,475,103-2,509,668, MIM: 613577)

TBC1 domain family member 24 *(TBC1D24*) is a member of the TBC domain-containing RAB-specific GTPase-activating proteins and is necessary for normal brain development via regulation of synaptic function and vesicle trafficking (104). There is marked phenotypic-pleiotropy associated with variants in *TBC1D24* including DEE, progressive myoclonic epilepsy, non-syndromic deafness, DOORS syndrome (deafness, onychodystrophy, osteodystrophy, mental retardation, and seizures), an AHC-like phenotype, multifocal myoclonus, epilepsia partialis continua or ID (41, 105–109). Inheritance is predominantly AR or compound heterozygous. However, AD inheritance is associated with the deafness phenotype.

Paroxysmal-exercise induced movement disorder (dystonia) is described in two children with onset in infancy with paroxysmal dystonic episodes, triggered by physical exertion and co-existing with epilepsy, ataxia, dysarthria and cerebellar dysfunction (6). There was a gradual improvement over time due to trigger avoidance. Also, paroxysmal facial and limb myoclonus with onset in early infancy, triggered by fever and fatigue has been published (42).

### *PDE2A* (Chr11:72,576,140-72,674,421, MIM 602658)

Biallelic LOF variants in phosphodiesterase 2a, cGMP-stimulated (*PDE2A*) is a newly reported gene associated with paroxysmal dyskinesia with developmental delay, chorea, ID and epilepsy. To date, only four patients (three families) are reported (7, 110). Thus, further expansion of this phenotype is needed.

## Predominant Episodic Ataxia Genes

Episodic ataxia is characterized by paroxysmal truncal ataxia, in-coordination, dysarthria and balance difficulties. Neurological interictal examination may be completely normal or may provide a clue to the underlying cause with myokymia (PxMD-*KCNA1*). Episodic ataxia was previously subdivided into nine subtypes depending on the underlying genetic cause. Episodic ataxia was common to all subtypes, but each had distinctive clinical characteristics. However, EA is now incorporated under PxMDs classification, due to the episodic nature of symptoms, overlapping clinical features and common genes with other paroxysmal disorders (12). For example, episodic ataxia type 1 is now referred to as PxMD-*KCNA1*. Table 2 provides a list of predominant episodic ataxia genes organized according to function detailing inheritance, phenotypes and potential treatments. We have chosen to discuss four EA genes in detail on the basis of their high prevalence in clinical practice in the case of two genes (*KCNA1, CACNA1A*) and the recent discovery and newly described phenotypes (*SLC1A3* and *FGF14*).

**Table 2:** Predominant episodic ataxia genes.

**Locus symbol/new designation (most common in bold)**	**Gene**	**Cytogenic location**	**Inheritance**	**Paroxysmal movement disorder (most common in bold)**	**Other associated phenotypes**	**Potential treatment**	**References**
**Neurotransmission and Synaptopathies**							
EA9/PxMD-*FGF14*	*FGF14*	13q33.1	AD	**EA** (Fever trigger ataxia) PNKD PKD	SCA 27 ID	ACZ	(16–21)
PxMD-*PRRT2*	*PRRT2*	16p11.2	AD/AR	EA **PKD** PED PNKD Paroxysmal torticollis	Absence epilepsy DEE FHM ID Self-limiting neonatal seizures	ACZ (EA) CBZ (PKD)	(4, 8, 27–37)
**Channelopathies**							
–	*ATP1A3*	19q13	AD	EA **AHC** PNKD PED	CAPOS DEE Rapid onset dystonia-parkinsonism	FLUN KD	(23, 45, 46)
**EA2/PxMD-*****CACNA1A***	*CACNA1A*	19p13.13	AD/AR	**EA** (Ataxia, dysarthria & nystagmus lasting hours. Interictal nystagmus) PED PNKD & epilepsy Paroxysmal torticollis Paroxysmal tonic upward gaze	Chronic ataxia DEE Epilepsy FHM ID SCA 6	ACZ 4-AP FLUN VPA	(47–54, 111–113)
EA5/PxMD-*CACNB4*	*CACNB4*	2q23.3	AD	EA (Ataxia, vertigo, dysarthria, & nystagmus, lasting hours. Interictal nystagmus & ataxia)	Epilepsy	ACZ	(18, 114, 115)
**EA1/PxMD-*****KCNA1***	*KCNA1*	12p13.32	AD	**EA** (Brief, daily episodes of ataxia & interictal myokymia) PKD PNKD	Epilepsy DEE Developmental delay & chronic ataxia Malignant hyperthermia	ACZ CBZ	(47, 111, 112, 116–118)
–	*KCNA2*	1p13.3	AD	EA & epilepsy	DEE & chronic ataxia	ACZ 4-AP	(119)
EA6/PxMD-*SLC1A3*	*SLC1A3*	5p13.2	AD	EA (Ataxia with vertigo, slurred speech, nausea. No interictal findings)	AHC Epilepsy ID Migraine	ACZ	(47, 120–123)
–	*NALCN*	13q32.3	AD	EA	ID Hypotonia Congenital contractures	ACZ	(124, 125)
**Brain energy transportopathies**							
PxMD*-SLC2A1*	*SLC2A1*	1p34.2	AD	EA **PED** PNKD PKD PED + spasticity PED + epilepsy	DEE Epilepsy/absence epilepsy FHM ID Spastic paraplegia	KD Trihepantoin	(4, 23, 71–77)
–	*BCKD complex*	19q13.2/6q14.1	AR	EA PNKD	MSUD	Isoleucine & valine restriction	(78)
–	*DARS2*	1q25.1	AR	Exercise-induced ataxia	Leukoencephalopathy with brain stem & spinal cord involvement	ACZ	(126)
**Pyruvate dehydrogenase complex**							
PxMD-*PDHA1*	*PDHA1*	Xp22.12	X-linked	PED PNKD Paroxysmal dystonia EA	Neonatal lactic acidosis Epilepsy Hypotonia Leigh Syndrome	Thiamine (PED/EA) Levodopa KD (Dystonia)	(82–85)
–	*PDHX*	11p13	AR				
–	*DLAT*	11q23.1	AR				
**Other**							
EA8/PxMD-*UBR4*	*UBR4*	1p36.13	AD	EA (Ataxia & slurred speech. Interictal ataxia, myokymia, nystagmus & tremor)	–	CLO	(18, 127)
EA3	Gene unknown	1q.42	AD	EA (Truncal ataxia, vertigo & tinnitus lasting <30 min. Interictal myokymia & nystagmus)	Epilepsy	ACZ	(114, 128)
EA4	Gene unknown	–	AD	EA (Ataxia, vertigo & diplopia. Interictal nystagmus)	–	–	(114)
EA7	Gene unknown	–	AD	EA (Ataxia, vertigo & dysarthria. No interictal features)	–	–	(114, 129)
**Candidate genes (recently described or described in a single family)**							
–	*CEP290*	12q21	AR	EA	Epilepsy Joubert syndrome	ACZ	(130)
–	*KCND3*	1p13.2	AD	EA	DEE SCA 19	–	(18, 131–133)
–	*TTBK2*	15q15.2	AD	EA	SCA 11	–	(18, 90)
–	*TGM6*	20p13	AD	EA	SCA 35	–	(18, 134)

*ACZ, Acetazolamide; AD, Autosomal Dominant; AR, Autosomal recessive; AHC, Alternating hemiplegia of childhood; CBZ, Carbamazepine; CLO, Clonazepam; DEE, Developmental and epileptic encephalopathy; EA, Episodic Ataxia; FHM, Familial Hemiplegic Migraine; FLUN, Flunarizine; ID, Intellectual Disability; JME, Juvenile myoclonic epilepsy; KD, Ketogenic diet, MSUD, Maple-syrup urine disease; OXY, Oxcarbazepine; PED, Paroxysmal Exercise Induced Dyskinesia; PKD, Paroxysmal Kinesigenic Dyskinesia; PMD, Paroxysmal movement disorder; PNKD, Paroxysmal Non-Kinesigenic Dyskinesia; SCA, Spinocerebellar ataxia; VPA, Sodium Valproate; 4-AP, 4-aminopyridine*.

### *KCNA1* (Chr 12:4,909,904-4,918,255, MIM: 176260)

Potassium channel voltage-gated shaker-related subfamily member 1 *(KCNA1)* encodes voltage-gated potassium channel (K_v_1.1), important in the repolarization of presynaptic action potentials that affect inhibitory input to Purkinje cells and cause hyperexcitability. All pathogenic variants are LOF, producing a markedly reduced inhibitory output of the cerebellum, thus causing symptoms (114). Inheritance is AD with reduced penetrance, intrafamilial and interfamilial phenotypical variability. Pathogenic variants are distributed throughout the gene in the EA phenotype, in contrast to the epilepsy phenotype where variants are limited to the pore domain. No genotype-phenotype correlations exist in the EA phenotype of *KCNA1* (111, 116, 135).

Pathogenic variants in *KCNA1* occur in up to 85% of EA type 1 phenotype, now referred to as PxMD*-KCNA1* (116). Episodic ataxia begins in childhood (mean age of onset is 7.8 years, range: 0–20 years), attacks are frequent and last for minutes characterized by poor coordination, loss of balance, tremor and slurred speech (116). There may be multiple daily attacks and recognized triggers include exertion, emotional stress and changes in environmental temperature. Myokymia (fine twitching or intermittent cramps and stiffness) is a persistent interictal feature, which is very suggestive of *KCNA1* but is recognized in other EAs including EA3 (no known causative gene, linked to cytogenic region Chr1q42) and PxMD-*UBR4* (previously called EA8). Over time, persistent cerebellar dysfunction occurs in a subset of patients. Episodic ataxia may be isolated or associated with epilepsy, epileptic encephalopathy, malignant hyperthermia and neuromyotonia (117). Epilepsy, cataplexy, PKD, PNKD, myokymia and migraine are reported in the absence of EA (47, 112, 118). Non-neurological presentations include hypomagnesaemia (111).

### *CACNA1A* (Chr19: 13,206,441-13,506,478, MIM: 601011)

Voltage-gated calcium channels are expressed throughout the central nervous system, mediating the entry of calcium into excitable cells. They are involved in multiple calcium-dependent processes, such as muscle contraction, neurotransmitter release or gene expression. Calcium channel voltage-dependent P/Q subtype, alpha 1-a subunit *(CACNA1A)* encodes the α_1_ subunit of voltage-gated P/Q calcium channel (Ca_v_2.1). Loss-of-function variants reduce calcium entry through Ca_v_2.1 resulting in irregular firing of the Purkinje and granule cells, where Ca_v_2.1 is largely expressed (114).

Inheritance is AD (penetrance 80–90%) and a single family with biallelic variants is reported (136). Gain-of-function variants are associated with FHM, epilepsy and DEE (137, 138). In contrast, LOF variants occur in PxMDs, most frequently in EA but also in PKD and PED. and trinucleotide CAG repeat expansion in spinocerebellar ataxia (SCA) type 6 (139, 140). Frequently, phenotypes may overlap with migraine reported in the EA phenotype, and EA symptoms in SCA6. Intrafamilial phenotypic variability includes cases of DEE, EA, ID and asymptomatic carriers all identified within one family (141).

The most common PxMD associated with LOF variants in *CACNA1A* is EA, less frequently PKD and PED. *CACNA1A-EA* is characterized by onset, from early childhood to early adulthood, of less frequent attacks than in PxMD*-KCNA1* (weekly or only a few episodes per year) with nystagmus, vertigo, nausea, vomiting and dysarthria, which last for hours. Interictal phenomena include nystagmus and migraine. Magnetic resonance imaging may demonstrate cerebellar atrophy, which may be static or progressive (139, 142). Migraine occurs in 50% of PxMD-*CACNA1A* cases (48, 114). Paroxysmal tonic up-gaze, nystagmus, and other eye movement disorders are all features of *CACNA1A* and present in up to 90% interictally (47). Eye movement disorders and paroxysmal tonic up-gaze may manifest in infancy before EA emerges years later (143). Variants in *CACNA1A* are associated with paroxysmal torticollis, paroxysmal benign tonic up-gaze, congenital cerebellar ataxia, autism spectrum disorder, DEE, chronic progressive ataxia, epilepsy and developmental delay (49–51, 142, 144). A severe phenotype of DEE, optic nerve atrophy and progressive cerebral and cerebellar atrophy with biallelic variants has been reported (136).

### *SLC1A3* (Chr 5:36,606,605-36,688,333, MIM: 600111)

The glial glutamate transporter excitatory amino acid transporter 1 (EAAT1) is present in the cerebellum and brainstem and encoded by solute carrier family 1, member 3 (*SLC1A3)*. EAAT1 is responsible for glutamate uptake in the synapses (114). Pathogenic variants in *SLC1A3* can cause both reduced and enhanced glutamate transport and thus alters channel activity. The cerebellum is sensitive to slight alterations in EAAT1 activity (145).

Heterozygous variants in *SLC1A3* are responsible for PxMD-*SLC1A3*, previously termed episodic ataxia type 6. Less is known about PxMD-*SLC1A3* than other EAs, with only 11 patients (seven families) reported to date (145). The onset of EA is variable from infancy to adulthood, and events are similar to PxMD-*CACNA1A* but longer in duration (hours to days) and there is no associated myokymia, nystagmus or tinnitus (146). Potential event triggers include emotional stress, physical exertion, caffeine and fever (47, 120). Additional features include seizures and migraine. Inheritance is AD. Duplications of *SLC1A3* cause developmental delay and behavioral problems in the absence of cerebellar findings (147).

### *FGF14* (Chr: 13:101,710,803-103,402,442, MIM: 601515)

Fibroblast growth factor 14 (*FGF14*) has been proposed to be added to the list of distinct EA phenotypes. To date, 12 patients (seven families) are published with an identical phenotype (16–20). Ataxia episodes are triggered by fever and can be associated with dysarthria, vertigo, headache and vomiting of variable duration and frequency. Episodes have a variable age of onset from early childhood to adulthood. Inter-ictal upper limb tremor, nystagmus and ID are present. Paroxysmal kinesigenic dyskinesia and PNKD are reported in isolation or associated with EA or SCA27 (148). Inheritance is AD with intrafamilial phenotypic heterogeneity (20).

Highly expressed in the brain, especially in granule and Purkinje cells, *FGF14* regulates pre-synaptic Ca_v_2.1 channel and vesicular recycling/synaptic transmission. *FGF14* may be a risk factor for neuropsychiatric disorders (depression, addiction) and neurodegenerative diseases and thus the phenotype continues to expand (149).

## Discussion

### History and Examination

Although we have summarized the expanding number of genes associated with PxMDs, it is still necessary to begin with the key clinical features as a guide to requesting and interpretation of genetic testing. Thus, a detailed history of the PxMD focusing on the phenomenology, triggers (movement, exercise, alcohol or fever) alleviating factors and the duration and frequency of attacks. In addition, information on other paroxysmal (hemiplegic migraine, epilepsy), and non-paroxysmal symptoms (ID, psychiatric co-morbidities etc.) should be sought (2, 150). Frequently the PxMD, may not fit into a distinct phenotype or may have features of different paroxysmal dyskinesias (for example both kinesigenic and exercise-induced features). A family history of PxMDs or other paroxysmal phenomena (migraine, infantile seizures) may be important because of interfamilial phenotypic heterogeneity and may aid interpretation of genetic findings.

In paroxysmal dyskinesia, examination is usually normal, and the presence of focal neurological deficits or upper motor neuron signs suggest a secondary causes of PxMDs. In EA, nystagmus, myokymia, upper limb tremor or chronic ataxia maybe a clue to the underlying causes (myokymia in PxMD-*KCNA1* and PxMD-*UBR4*).

### Approach to Evaluating a Patient With Paroxysmal Movement Disorder

**Figure 1:**
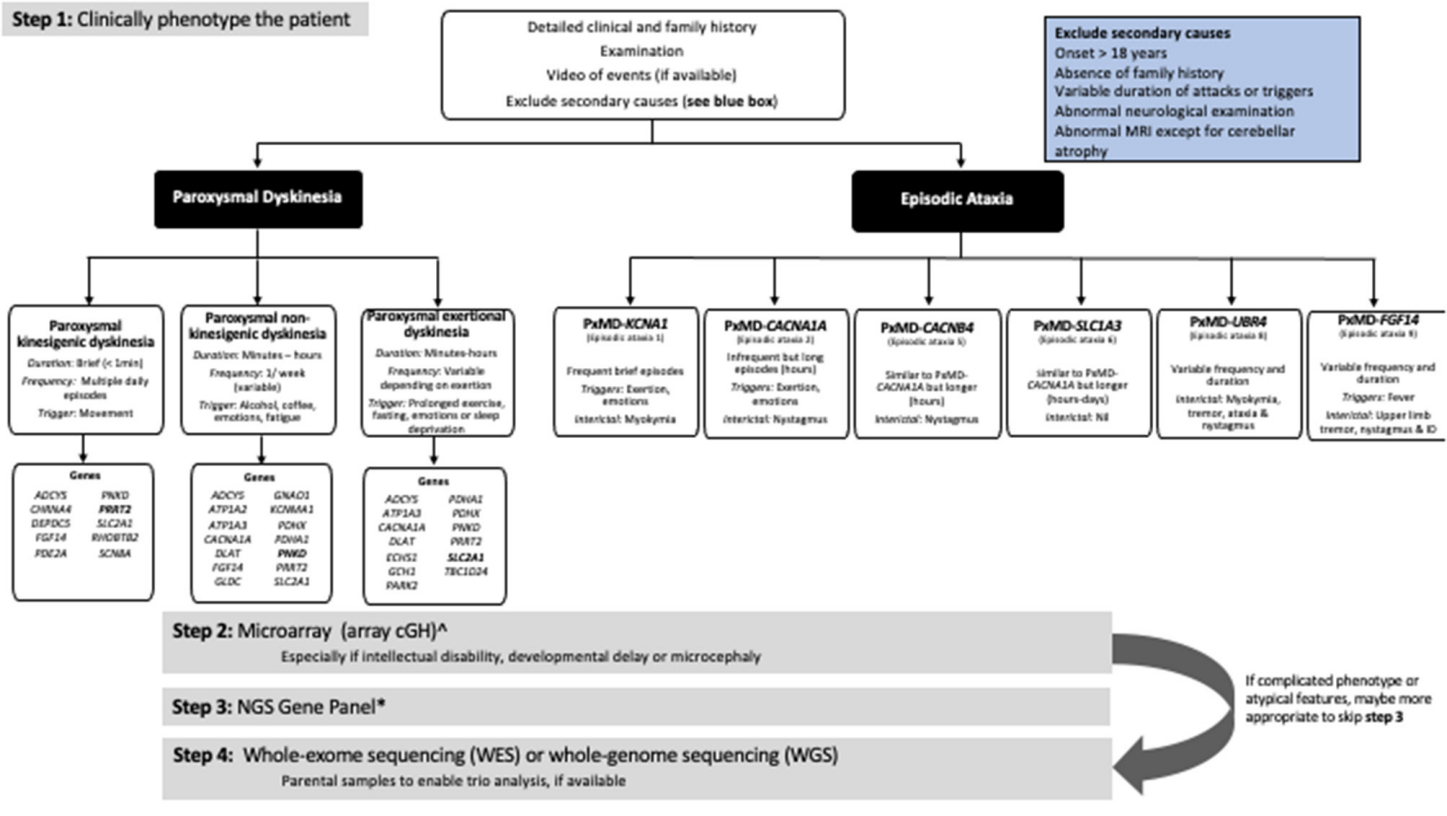
Approach to the genetic investigation of paroxysmal movement disorders. ^∧^Will not be required if using whole-genome sequencing. *The number of genes varies depending on the NGS panel used. Always check what genes are included in the panel.

Before devising an investigation strategy, a thorough history and examination are essential, to determine whether or not the PxMD is primary or secondary and to define the main clinical features of the PxMD, as this will dictate how to approach investigations. Secondary or acquired causes of PxMDs, should be excluded clinically or with appropriate metabolic or radiological studies. Red flags for secondary causes of PxMDs include onset in adulthood, absence of family history, variable duration of attacks and triggering factors, progressive disease course, abnormal inter-ictal neurological examination (dystonia, upper motor neuron signs) or abnormal magnetic resonance imaging (Figure 1).

### Diagnostic Yield in Paroxysmal Movement Disorders

In the past 20 years, there has been a rapid advance in the understanding of the genetic basis of neurological disorders, including PxMD. However, many patients with the typical clinical features of PxMD remain without a genetic diagnosis. When NGS is utilized, the diagnostic yield is only 35–50% (Supplementary Table 1). The yield is equal or less compared to other pediatric-onset neurological disorders such as epilepsy (up to 70%), leukodystrophy (up to 90%), mixed movement disorder (51%), childhood-onset ataxia (46%) or progressive ataxia (32–53%) (87, 151–158).

### Algorithm for Investigation

Traditionally in PxMDs, sequential single-gene testing was performed based on the predominant movement. For example, in PKD, single-gene testing of *PRRT2* was performed, followed by *SLC2A1* and *PNKD*. Diagnostic rates were <50% when the three genes were sequenced in a cohort with paroxysmal dyskinesias and EA (4). However, as highlighted in this article due to phenotypic pleiotropy, single-gene testing is no longer the best approach.

A microarray (arrayCGH) should be the first tier of genetic testing and is particularly relevant if epilepsy, ID or developmental delay is present (159–161). Diagnostic yield of microarray in pediatric movement disorders is reported in a single center cohort (*n* = 20) only. Pathogenic copy number variants (CNVs) were detected in 28% (7/20) in a heterogenous group with movement disorder and 40% (2/5) in PKD (162). The yield in children with undiagnosed neurological conditions ranges from 8.9 to 28%, depending on the cohort (163–165). Microarray should be requested even if a NGS gene panel or WES is requested as these technologies may not detect CNVs (158).

Due to genetic heterogeneity, gene-panel testing is recommended as second-tier testing, enabling parallel sequencing of multiple genes. The number of genes on NGS panels is variable, depending on the individual laboratory and when the test was performed. Gene panels for PxMDs are found under an assortment of different titles, including paroxysmal disorders, degenerative disorders, channelopathies or episodic disorders. A search of commercially available panels in European laboratories highlighted the number of genes tested varied between 6 and 186 genes, depending on the laboratory (Supplementary Table 2). Therefore, knowledge of the specific panel requested is essential as key genes and recently described genes may be missed or not included at the time of testing. For example, *TBC1D24*, may only be available on an epilepsy panel. An advantage of a panel approach over clinical whole-exome sequencing (WES) is that many panels include high-resolution coverage, including for exon-level deletions or duplications, which are not detected by standard sequencing techniques. Also, panel analysis rather than WES/WGS reduces the possibility of secondary reportable findings and variants of unknown significance not related to the phenotype (166).

In cases where there is a complicated clinical phenotype, WES or whole-genome sequencing (WGS) should be employed as second-tier genetic testing. If panel testing is negative, then WES or WGS may be requested. Both WES and WGS will enable identification of new genes, expansion of phenotype of known genes and recognition of new clinical phenotypes. There is a paucity of studies on the yield of WES/WGS in PxMDs (Supplementary Table 1); the majority of publications to date utilized a panel analysis of WES data (18, 69, 151, 167, 168). In the near future, WGS is likely to be the most cost and time-efficient approach to investigating patients (169).

### Limitations of NGS Technology

Next-generation sequencing has revolutionized how we approach genetic-testing but the technology has several limitations. Both WES and WGS, cannot detect balanced translocations, variants are missed due to low coverage or poor sequence quality in G-C rich regions, difficulty in interpretation of short or long insertions and WES cannot detect large deletions/duplications (CNVs). Whole-genome sequencing provides advantages over WES notably continuous coverage, the ability to identify sequence variants throughout the genome (including intronic variants, intergenic and regulatory sequence variants, expanded repeats) and to detect smaller CNVs than detected on microarray (158, 170–172). However, while deep intronic, noncoding and intergenic variants are all potentially detectable by WGS they can be challenging to interpret due to the lack of functional protein to validate findings (166, 172). Triplet repeat expansions analysis is possible with WGS, but many bioinformatic pipelines are still only available on a research basis and require long-read NGS (172–174). Thus, if a trinucleotide repeat disorder is suspected, this needs to be specifically requested additional to WES analysis. High coverage of PxMDs genes is required to detect mosaic variants (174).

### Mosaicism

Mosaicism is increasingly recognized within pediatric neurology as a pathogenic mechanism in monogenic disorders (~3% in neurodevelopmental disorders) (175, 176). In PxMDs, mosaic variants have been reported in *ATP1A3, ADCY5, SLC2A1 and PDHA1*, (98, 177–179). In *ADCY5*, mosaicism is reported in 25–50% of cases, associated with a milder phenotype and symptoms may not be recognized (96, 98). Parental mosaicism, if undetected, impacts on genetic counseling and risk for future pregnancy. Good read depth (>100 for WES and >30 for WGS), modification of filters/algorithms, sequencing of additional tissue or single-molecule molecular inversion probes may be required to detect somatic mosaicism (176).

### Interpretation of Genetic Findings

The correct interpretation of results from microarray, gene panels and WES/WGS is essential, particularly when a novel variant is identified (180). Inheritance studies for segregation within families is helpful to support pathogenicity. However, with PxMDs reduced penetrance and variable phenotypes within families add to the complexity when evaluating findings. Functional studies and/or identification of variants within multiple individuals are needed to apply correct pathogenicity to new variants. Finally, clinical-genetic expertise is crucial for clinical phenotyping, evaluating and interpreting the genetic results, and communication with the patient and family.

### Importance of Genetic Testing

The importance of identification of a genetic cause cannot be overstated. The identification of the correct genetic diagnosis enables accurate genetic counseling (including preimplantation genetic diagnosis, where applicable), prediction of disease phenotype, precision treatment strategies (the ketogenic diet in PxMD-*SLC2A1)*, surveillance of associated co-morbidities, and engagement in family support groups (2, 181, 182). Finally, an earlier genetic diagnosis avoids invasive and expensive investigations such as muscle biopsy, multiple hospital visits and missed days from school/work (169).

### Future Directions

Though new genes associated with PxMDs have been discovered, only 50% of individuals with PxMDs have an underlying genetic diagnosis. Therefore, further work is needed to elucidate a cause for the remainder. Episodic ataxia types 3 and 7 are only linked to chromosomal regions (EA3 linked to 1q42, EA7 to 19q13) and EA4 is not linked to a known locus. However, a single gene has not been identified in these regions to explain the phenotype. Adults with symptom onset in childhood should have previous genetic testing re-evaluated in light of the evolution of NGS and the discovery of new genes in the last decade. Re-interrogation of WES and WGS data for “negative patients,” with time is necessary. Several groups have demonstrated the increased yield of 5.8% in DEE and 7% in ataxia (157, 183, 184) after re-analysis.

As discussed in the limitations paragraph, WGS provides advantages over WES/gene panel analysis with better coverage, additional analysis including repeat expansions, CNV analysis and identification of deep intronic variants (166). The application of transcriptome analysis/transcriptome data to WGS findings improves the diagnosis of WGS findings by aiding the interpretation of variants. This has already been employed in neuromuscular disorders with an increased diagnostic yield (166, 185).

Additional features must contribute to the clinical presentation of PxMDs given the infrafamilial heterogeneity and reduced penetrance. Epigenetics refers to cellular processes that influence gene expression outside of direct changes to the DNA and includes methylation, histone modification, microRNAs, transcriptome and the microbiome (158, 186–189). Further work is needed to fully understand the role of epigenetics in PxMDs (187).

### Impact of Treatment

Discussion regarding specific treatment of PxMDs is beyond the scope of this review and is discussed in detail elsewhere (2, 190). Identification of a genetic cause is the first step toward the development of precision therapy targeting specific pathophysiological mechanisms and potential gene therapy for PxMDs (181). Currently, treatment is often based on the clinical phenotype rather than genotype as in the use of carbamazepine in PKD. Despite small numbers reported with certain pathogenic variants, patterns are emerging, showing better efficacy of some medications in certain genotypes. For example, in *SCN2A* GOF variants respond to sodium channel blockers which are ineffective in patients with LOF variants (191). Thus, knowledge of the causative variants in successfully treated patients will aid the treatment approach in the future.

## Conclusion

Paroxysmal movement disorders are a genetically heterogeneous group of movement disorders with phenotypic pleiotropy. Next-generation sequencing has revolutionized how we classify and investigate PxMDs. However, a large proportion of patients remain undiagnosed compared to other neurological diseases. Thus, further research is needed in PxMDs to identify new genes, understand disease mechanisms and develop precision treatments.

## Author Contributions

SH, MK, and KG contributed to the design, writing, and review of the manuscript. All authors contributed to the article and approved the submitted version.

## Conflict of Interest

The authors declare that the research was conducted in the absence of any commercial or financial relationships that could be construed as a potential conflict of interest.

## Abbreviations

AD: Autosomal dominant
AHC: Alternating hemiplegia of childhood
AR: Autosomal recessive
DEE: Developmental and epileptic encephalopathy
GLUT-1: Glucose-transport-1
EA: Episodic Ataxia
FHM: Familial hemiplegic migraine
GOF: Gain-of-function
ID: Intellectual disability
LOF: Loss-of-function
NGS: Next-generation sequencing
PED: Paroxysmal exercise induced dyskinesia
PKD: Paroxysmal kinesigenic dyskinesia
PxMD: Paroxysmal movement disorders
PNKD: Paroxysmal non-kinesigenic dyskinesia
SCA: Spinocerebellar ataxia
WES: Whole-exome sequencing
WGS: Whole-genome sequencing.
