# Supplementary material for: Paroxysmal Movement Disorders
Source: Front Neurol. 2021 Jun 11;12:659064. doi: 10.3389/fneur.2021.659064 (PMC8232056; doi:10.3389/fneur.2021.659064)
Supplement: Supplementary file 1 [file Table_1.DOCX]

# **Supplementary Table 1: Summary of publications on the yield of NGS in paroxysmal movement disorders**

| Paper | NGS Platform | Number of genes | Phenotype (numbers) | Yield | Reference |
| --- | --- | --- | --- | --- | --- |
| Maksemous et al., 2020 | WES – Gene panel analysis | 173 (ataxia)  353 (ion channel) | EA2 (16) – all *CACNA1A negative* | 8/16 (50%) Genes: *SCN2A, ATP1A3, KCNA1, PEX7* Candidate genes: *CACNA1E, SCN1B, SCN9A, CLCN1* | (1) |
| Graziola et al., 2019 | Gene Panel | 102 (MD genes) | Movement disorders (134)  PxMD (34) EA (10) PKD (18) PED (2) Hemiplegic attacks (4) Paroxysmal myoclonus (1) | Overall yield: 12/34 (35%)  10% PKD 10% EA 50% PED (n=1) 25% Hemiplegic attacks | (2) |
| Montaut et al., 2019 | Gene panel | 127 | Movement disorder (378) PxMD = 20 | 7/20 with PKD (35%) Overall yield = 22% | (3) |
| Tian et al., 2018 | WES – Gene panel analysis | 27 | PKD (163) -all *PRRT2* negative | 10/136 (6 families) (7.4%) | (4) |
| Choi et al., 2017 | WES – Gene panel analysis | 40 | EA | 18/39 (46%) (11 pathogenic and 7 likely pathogenic variants) | (5) |
| Gardiner et al., 2015 | *-* | Sequencing of 3 genes | PxMD (145) EA and FHM (53) | PxMD (47%) *PRRT2* 35% *SLC2A1* 10% PNKD 10%  EA/FHM (53) 2 *PRRT2* 1 *SLC2A1* 1 *PNKD* | (6) |
| **Abbreviations:** EA, Episodic Ataxia; FHM, Familial Hemiplegic Migraine; PED, Paroxysmal Exercise Induced Dyskinesia; PKD, Paroxysmal Kinesigenic Dyskinesia; PNKD, Paroxysmal Non-Kinesigenic Dyskinesia; PxMD, Paroxysmal Movement Disorder; WES, Whole-exome sequencing | | | | | |

| **Supplementary Table 2: Currently available gene panels in Europe for Paroxysmal Movement Disroders** | | | |
| --- | --- | --- | --- |
| Laboratory Name | Gene Panel Title | Number of genes | List of genes |
| CeGat GmbHTübingen,Germany | Paroxysmal movement disorders(NDD08) | 15 | *ADCY5, ATP1A2, ATP1A3, CACNA1A, GCH1, KCNA1, KCNMA1, NOTCH3, PNKD, POLG, PRKN, PRRT2, SCN1A, SCN8A, SLC2A1* |
| CeGat GmbHTübingen,Germany | Episodic Ataxia(NDD30) | 6 | *CACNA1A, CACNB4, FGF14, KCNA1, SCN2A, SLC1A3* |
| CentogeneRostock,Germany | Ataxia Panel | 186 | *BCB7, ABHD12, ABHD5, ACADVL, ACO2, AFG3L2, AHI1, ALDH5A1, AMACR, ANO10, AP1S2, APTX, ARL13B, ARL6, ARSA, ATCAY, ATM, ATN1, ATP13A2, ATP1A3, ATP2B3, ATP8A2, B9D1, BBS1, BBS12, BSCL2, BTD, C12orf65, C19orf12, CA8, CACNA1A, CACNB4, CAMTA1, CASK, CC2D2A, CCDC88C, CEP290, CEP41, CHMP1A, CLCN2, CLN5, CLN6, CLPP, COASY, COQ2, COQ8A, COQ9, COX20, CP, CPLANE1, CSPP1, CWF19L1, CYP27A1, DARS2, DLAT, DNAJC19, DNAJC5, DNMT1, EIF2B1, EIF2B2, EIF2B3, EIF2B4, EIF2B5, ELOVL4, ELOVL5, EXOSC3, FA2H, FBXL4, FGF14, FLVCR1, FTL, FXN, GALC, GBA, GBA2, GFAP, GJB1, GJC2, GOSR2, GRID2, GRM1, GSS, HEPACAM, HEXB, HIBCH, INPP5E, ITM2B, ITPR1, KCNA1, KCNC3, KCND3, KCNJ10, KIF1A, KIF1C, KIF5A, KIF7, LAMA1, LMNB1, LRPPRC, MARS2, MKS1, MLC1, MRE11, MTFMT, MTPAP, MTTP, NDUFAF6, NDUFS1, NDUFS2, NDUFS4, NDUFS7, NDUFV1, NPC1, NPC2, NPHP1, NUBPL, OFD1, OPA1, OPA3, OPHN1, PANK2, PAX6, PDHX, PDSS1, PDSS2, PDYN, PEX10, PEX2, PEX7, PHYH, PLA2G6, PLP1, PNKD, PNKP, PNPLA6, POLG, POLR3A, POLR3B, PRICKLE1, PRKCG, PRRT2, RARS2, RPGRIP1L, RRM2B, RUBCN, SACS, SCN2A, SETX, SIL1, SLC16A2, SLC17A5, SLC1A3, SLC20A2, SLC25A46, SLC2A1, SLC52A3, SLC9A6, SPG11, SPG7, SPR, SPTBN2, STUB1, SYNE1, TCTN2, TGM6, TMEM216, TMEM237, TMEM240, TMEM67, TPP1, TSEN2, TSEN34, TSEN54, TTBK2, TTC19, TTPA, TUBB4A, TWNK, UBA5, VAMP1, VLDLR, VRK1, WDR81, WFS1, WWOX, ZFYVE26* |
| Institute of NeurologyQueen Square,London,UK | Brain Channel NGS Panel | 11 | *ATP1A2 ATP1A3 CACNA1A CACNB4 KCNA1 KCNK18 PNKD PRRT2 SCN1A SLC1A3 SLC2A1* |
| MGZ,  Medical Genetics Center,  Munich  Germany | Dyskinesia, paroxysmal/dystonia | 6 | *ADCY5, DLAT, KCNMA1, PDHA1, PDHX, PNKD* |
| Cervco, Laboratoire de Génétique Moléculaire, Hopital Saint Louis, Paris France | Ataxie Episodique | 8 | *CACNA1A, KCNA1, CACNB4, SLC1A3, FGF14, PRRT2, GLUT1, ATP1A3* |
| Cervco, Laboratoire de Génétique Moléculaire, Hopital Saint Louis, Paris France | Mouvements anormaux paroxystiques | 8 | *PRRT2, PNKD, GLUT1, ATP1A3, ADCY5, SCN8A, GCH1, KCNMA1* |
| Asper Biogene  Tartumaa,  Estonia | Paroxysmal Dyskinesia NGS Panel | 5 | *ADCY5, KCNMA1, PNKD, PRRT2, SLC2A1* |
| University  Medical Center Utrecht,  The Netherlands | Epilepsy with paroxysmal disorders | 11 | *ATP1A2, ATP1A3, CACNA1A, KCNA2, KCNMA1, PRRT2, SCN1A, SCN8A, SLC1A3, SLC2A1, CTNND2* |
| Sheffield Diagnostic Genetic Service  United Kingdom | Episodic Ataxia | 6 | *CACNA1A, CACNB4, KCNA1, PRRT2, SLC1A3, SLC2A1* |

**References:**

1. Maksemous N, Sutherland HG, Smith RA, Haupt LM, Griffiths LR. Comprehensive Exonic Sequencing of Known Ataxia Genes in Episodic Ataxia. Biomedicines. 2020;8(5).

2. Graziola F, Garone G, Stregapede F, Bosco L, Vigevano F, Curatolo P, et al. Diagnostic Yield of a Targeted Next-Generation Sequencing Gene Panel for Pediatric-Onset Movement Disorders: A 3-Year Cohort Study. Front Genet. 2019;10:1026.

3. Montaut S, Tranchant C, Drouot N, Rudolf G, Guissart C, Tarabeux J, et al. Assessment of a Targeted Gene Panel for Identification of Genes Associated With Movement Disorders. JAMA Neurol. 2018;75(10):1234-45.

4. Tian WT, Huang XJ, Mao X, Liu Q, Liu XL, Zeng S, et al. Proline-rich transmembrane protein 2-negative paroxysmal kinesigenic dyskinesia: Clinical and genetic analyses of 163 patients. Mov Disord. 2018;33(3):459-67.

5. Choi KD, Kim JS, Kim HJ, Jung I, Jeong SH, Lee SH, et al. Genetic Variants Associated with Episodic Ataxia in Korea. Sci Rep. 2017;7(1):13855.

6. Gardiner AR, Jaffer F, Dale RC, Labrum R, Erro R, Meyer E, et al. The clinical and genetic heterogeneity of paroxysmal dyskinesias. Brain. 2015;138(Pt 12):3567-80.
